# Restoring chloride efflux in cystic fibrosis with TMEM16a antisense oligonucleotides

**DOI:** 10.1016/j.ymthe.2025.08.045

**Published:** 2025-09-08

**Authors:** Christie Mitri, Nathalie Rousselet, Pauline Bardin, Madara Dias Wickramanayaka, Tobias Foussignière, Gabrielle Dupuis, Marion Leblanc, Victoire Gournet, Florence Sonneville, Harriet Corvol, Olivier Tabary

**Affiliations:** 1 Sorbonne Université, INSERM, Centre de Recherche Saint-Antoine, CRSA, 75012 Paris, France; 2 Département de Pédiatrie Respiratoire, Hôpital Trousseau, AP-HP, Paris, France

**Keywords:** preclinical studies, lung, cystic fibrosis, CFTR therapy, anoctamin-1, TMEM16a, oligonucleotides, target site blocker, mRNA therapy

## Abstract

Patients with cystic fibrosis (CF) who are non-responsive to treatments due to specific mutations need alternative CF transmembrane conductance regulator (CFTR)-independent therapies. This study aims to assess the impact of TMEM16a expression by a specific oligonucleotide (TMEM16a ASO) on dysregulated parameters in CF, which will help prepare for preclinical studies. In this study, we analyzed the effects of TMEM16a oligonucleotide within a CF context by evaluating the impact, optimal administration route, toxicity, and specificity in primary cells and various mouse models. The oligonucleotide enhances TMEM16a activity, increases Cl^−^ efflux, and improves mucociliary clearance in cells from all individuals tested with CF. The TMEM16a antisense oligonucleotide (ASO) effect is detectable in mice 30 days after subcutaneous injection, enhances TMEM16a mRNA expression, and significantly extends the lifespan of CF mice. Acute administration of 50 times the effective dose showed no toxicity. Importantly, TMEM16a ASO is highly specific, not inducing inflammation or altering intracellular calcium mobilization and cell proliferation, instilling confidence in its potential effectiveness. Our study demonstrates that TMEM16a ASO can compensate for CFTR deficiency in CF models. Additionally, it is important to note that this strategy could apply to all CF patients, regardless of their *CFTR* mutation, thereby broadening the scope of treatment options for CF.

## Graphical abstract

**Figure:**
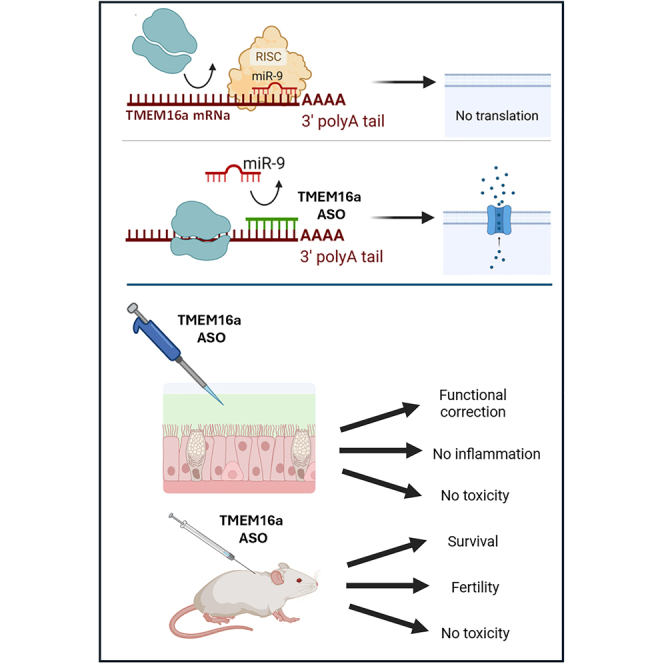

## Teaser

TMEM16a antisense oligonucleotides restore chloride efflux in cystic fibrosis (CF) models, boosting mucociliary clearance and increasing lifespan. This CF transmembrane conductance regulator-independent method provides a possible treatment for all CF patients, regardless of mutation type, by targeting the calcium-activated chloride channel TMEM16a.

## Introduction

Cystic fibrosis (CF) is an autosomal recessive genetic disease caused by mutations in the CF transmembrane conductance regulator (*CFTR*) gene.^1^ More than 2,100 mutations affecting protein production or activity have been identified. In CF, mutations are categorized into different classes based on their effects on CFTR protein function, ranging from class I mutations, which result in the absence of protein production, to class VI mutations, characterized by the production of a protein with a modified function. In CF, impaired Cl^−^ ion efflux coupled with sodium (Na^+^) ion hyperabsorption results in epithelial surface dehydration and the production of persistent, thick mucus that obstructs the lungs, pancreatic ducts, biliary tract, intestines, and male reproductive tract.^1^ The most common clinical manifestations of CF include chronic lung infections and pulmonary inflammation, which result in progressive tissue damage and loss of function, leading to increased morbidity and mortality.

Over the past decade, new treatments have revolutionized CF clinical management by introducing innovative therapies that target and activate CFTR at the apical membrane, especially with elexacaftor/tezacaftor/ivacaftor (ETI).^2^ Unfortunately, these approaches are mutation dependent and still not effective for 10%–15% of people with CF due to class I mutations or drug intolerance.^3^ Therefore, it is necessary to develop alternative therapies that are CFTR independent to enhance chloride activity and/or improve mucociliary clearance. Sodium channel (ENaC) blockers have been under development for some time to decrease ENaC hyperactivity in CF, although none have yet progressed through pivotal trials to licensing.^4^

In 2008, three research teams identified TMEM16a (anoctamin-1 [ANO1]) as the gene encoding the calcium-activated chloride channel (CaCC),^5^^,^^6^^,^^7^ and it has been proposed as an alternative strategy to compensate for CFTR deficiency.^8^ Therefore, a strategy to enhance TMEM16a expression or activity could bypass CFTR deficiency and benefit all people with CF (pwCF), especially those ineligible for current curative treatments. This strategy is highly controversial because multiple studies indicate that overexpression may play a role in regulating mucus secretion and is present in many types of cancer, including those of the digestive system such as esophageal squamous cell carcinoma and gastric, colorectal, and pancreatic cancers.^9^^,^^10^^,^^11^ Therefore, our primary approach is not to excessively express TMEM16a but to return its activity to normal levels, which have been disrupted in pwCF. We initially demonstrated the specific inhibitory role of microRNA-9 (miR-9) in regulating TMEM16a expression using a luciferase activity assay in the presence of a mimic or antagomiR, a finding that other research groups across various pathologies have since corroborated.^12^^,^^13^^,^^14^^,^^15^

Building on this insight, we developed a targeted approach based on antisense oligonucleotides (ASOs) to specifically prevent only miR-9 from binding to the 3′ UTR of TMEM16a mRNA through competitive interaction, thereby enhancing translation and its expression.^16^ ASOs are synthetically modified single-stranded nucleic acid sequences, typically ranging from 12 to 30 nt in length, that selectively bind to specific complementary RNA targets through Watson-Crick base pairing.^17^ The sequence of TMEM16a ASO was designed to be specific to human and mouse sequences. Locked nucleic acids (LNAs) and phosphorothioate modifications were introduced to enhance the stability, affinity, and specificity of the oligonucleotides. We selected this strategy due to the reported benefits of ASO: specificity, versatility, and therapeutic potential. In this article, we strengthened the initial observation published on F508del cells by incorporating additional cell models, electrophysiological data, and toxicity studies, and we broadened this research to include various cells and mice with CFTR class I mutations.

Our research suggests that this strategy can be implemented in pwCF to restore Cl^−^ efflux and mucociliary clearance without causing inflammation or toxicity. Furthermore, our research has demonstrated that this strategy can treat various organs affected by CF, increasing the life expectancy of CF mice and enhancing the fertility of CF male mice. Our findings support the concept that an increase in TMEM16a Cl^−^ activity may represent a promising avenue for drug development for all pwCF.

## Results

### TMEM16a ASO potentiates CaCC chloride efflux in class II CF cells

**Figure 1:**
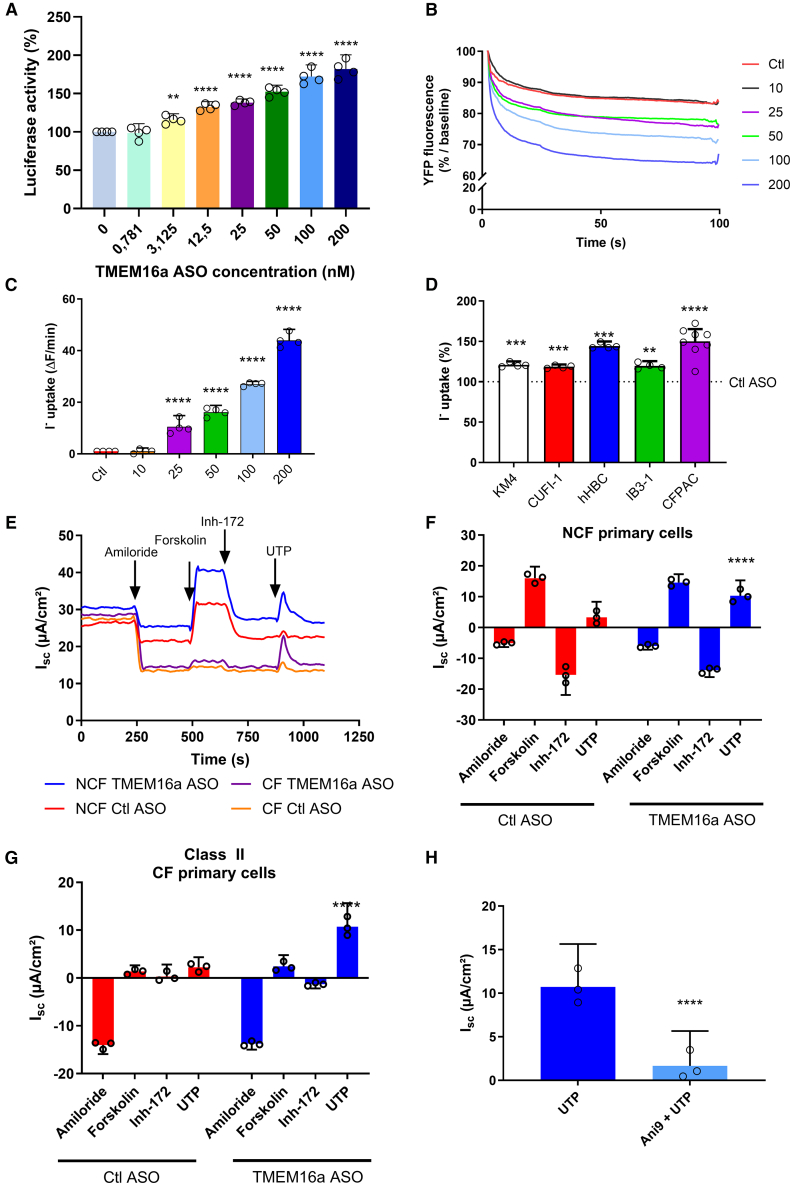
TMEM16a ASO increases Cl^−^ efflux in F508del cells (A) Relative luciferase activity in HEK cells stably transfected with a luciferase-3′ UTR ANO1 and treated with TMEM16a ASO at the indicated concentration. Firefly luciferase activity was normalized to Renilla luciferase activity (*n* = 4, with 8 replicates). (B) Representative curve of Cl^−^ efflux assessed by halide-sensitive YFP probe in CFBE41o^−^ cells transfected with control ASO (Ctl ASO) or TMEM16a ASO for 24 h at the specified concentrations in nanomoles. (C) Quantification of iodine (I^−^) assessed by halide-sensitive YFP probe in CFBE41o^−^ cells transfected with Ctl ASO or TMEM16a ASO for 24 h at the specified concentrations in nanomoles (*n* = 4). (D) Quantification of iodine (I^−^) uptake in (C) (*n* ≥ 4). (E) Representative traces of electrophysiological responses in Ussing chambers of non-CF and CF (F508del/F508del) hBECs transfected with Ctl ASO or TMEM16a ASO for 1 h/day over 3 days at 50 nM. Black arrowheads indicate the addition of amiloride to block sodium channels (100 μM), forskolin to stimulate CFTR (10 μM), or UTP to activate the purinergic calcium-dependent Cl^−^ secretion (100 μM). CFTR was inhibited with CFTR inhibitor 172 (CFTRinh-172) (10 μM). (F) The normalized total area under the curves of Ussing chamber assays of CF hBEC cells activated by UTP and pretreated with Ani9 (10 μM) or the vehicle (*n* = 3). Quantification of the normalized total area under the curves of Ussing chamber assays of CF bronchial primary cells (*n* = 3) before (G) and after (H) Ani9 treatment (10 μM, 1 h). Data are presented as mean ± SD with a 95% confidence interval. ANOVA followed by Dunnett’s and Bonferroni’s post hoc tests. ∗∗*p* ≤ 0.01; ∗∗∗*p* ≤ 0.001; ∗∗∗∗*p* ≤ 0.0001.

To determine whether TMEM16a ASO induces TMEM16a expression by binding to the TMEM16a 3′ UTR, HEK cells were stably transfected with a luciferase reporter vector containing the TMEM16a 3′ UTR sequence. Cells were treated for 24 h with TMEM16a ASO at the specified concentration. The results confirmed that under basal conditions, TMEM16a increases the activity of the 3′ UTR TMEM16a, as visualized by the expression of luciferase (Figure 1A). Initially, TMEM16a chloride activity was assayed using a halide-sensitive yellow fluorescent protein (YFP) probe. We demonstrated that TMEM16a ASO greatly enhanced the TMEM16a Cl^−^ channel activity in a dose-dependent manner compared to the control (Ctl) ASO, starting at 25 mM (Figures 1B and 1C). These results were confirmed using various F508del cell types, including tracheal CF gland serous cells (KM4), bronchial epithelial cells (CUFI and IB3-1), primary human bronchial glandular cells (hBGC), and pancreatic cell line (CFPAC) (Figure 1D). Compared to the cells treated with Ctl ASO, the effects observed in Cl^−^ efflux assays are significant across all tested CF cells, even though the degree of correction varies. In CF cells treated with TMEM16a ASO, the Cl^−^efflux increased by 20%–25% in KM4, CUFI, and IB3-1, reaching nearly 50% in the most responsive cells, such as hBGC and CFPAC (Figure 1D). These results appear proportional to the basal expression of TMEM16a in the various cells tested (data not shown).

The transepithelial Cl^−^ current, driven by cyclic AMP (cAMP for CFTR or Ca^2+^-mediated signaling pathways for TMEM16a, was measured using the Ussing chamber method. In primary human bronchial epithelial cells (hBECs), we observed a strong CFTR-dependent Cl^−^ efflux in non-CF hBECs (Figures 1E–1G). This activation was abolished by the specific FTR inhibitor Inh-172 compared to CF hBEC cells. Uridine-5′-triphosphate (UTP)-induced Ca^2+^-mediated Cl^−^ efflux was significantly higher in both ASO TMEM16a-treated CF and non-CF cells compared to the control group (Figures 1E–1G). The TMEM16a-mediated Cl^−^ efflux was strongly inhibited by the specific inhibitor of TMEM16a, Ani9,^18^ demonstrating the specificity of the TMEM16 ASO activation (Figure 1H).

### TMEM16a ASO increases Cl^−^ activity in class I CF cells

**Figure 2:**
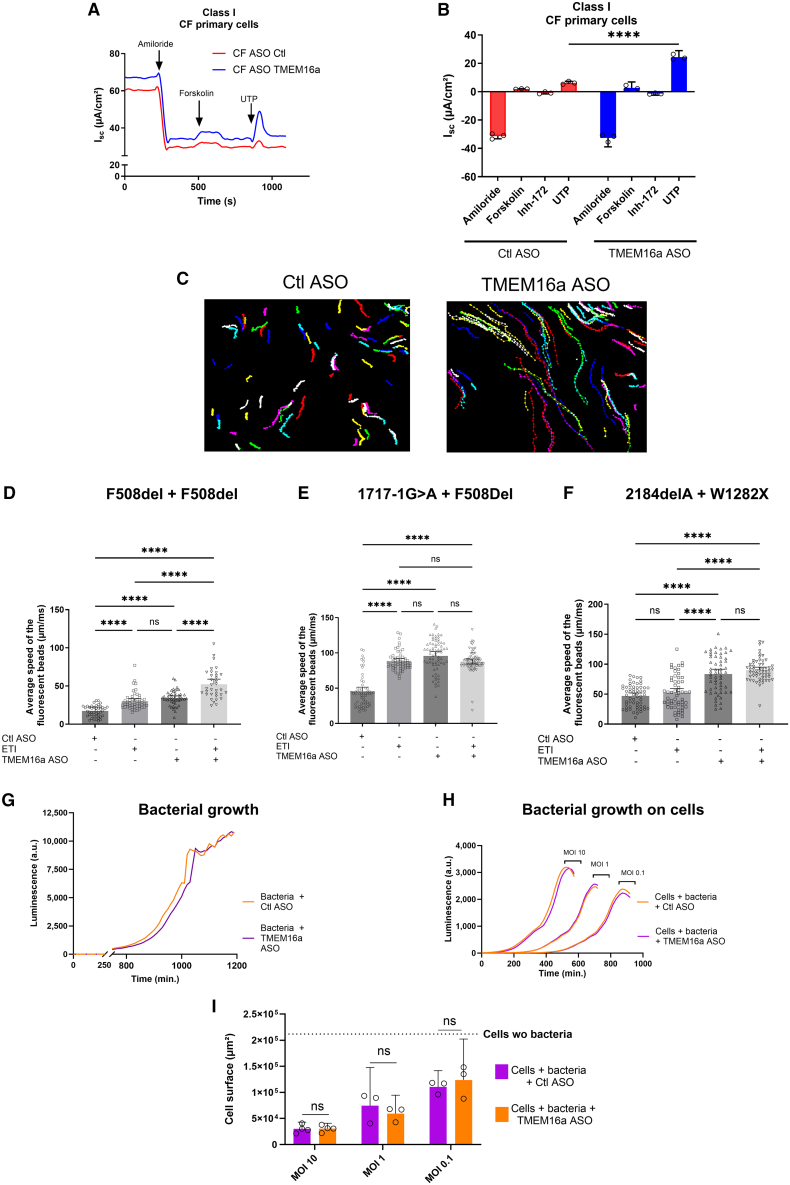
TMEM16a ASO enhances the UTP Cl^−^ response and mucociliary clearance across various cells with different CFTR mutations while not impacting other dysregulated parameters of CF (A) Representative Ussing experiments of non-CF and CF hBEC transfected with Ctl ASO or TMEM16a ASO for 1 h/day for 3 days at 50 nM. Black arrowheads indicate the addition of amiloride, forskolin, CFTR inhibitor 172, or UTP during the experiment. (B) Quantification of the normalized total area under the curves of Ussing chamber assays of CF hBEC (2184DelA W1282x). Data are presented as mean ± SD (*n* = 3). (C) Representative examples of fluorescent bead tracking on Ctl or TMEM16 ASO-treated hBEC (F508del/F508del). Scale bar: 100 μm. (D–F) Quantification of average bead velocity measured in hBEC with (D) F508del/F508del, (E) 1707-1G>A/F508del, and (F) 2184DelA/W1282x mutations following the specified treatments (*n* = 5 per condition, with 100 beads tracked per sample). (G) Representative curve of bioluminescence production and bacterial growth over time for *P. aeruginosa* cultured alone with Ctl or TMEM16a ASO (50 nM, *n* = 5). (H) Representative curve of bioluminescence production and bacterial growth over time for *P. aeruginosa* cultured at MOIs of 0.1, 1.0, or 10 bacteria/cell, in the presence of CFBE41o^−^ pretreated with either Ctl or TMEM16a ASO for 1 h/day for 3 days at 50 nM (*n* = 5). (I) Quantifying infected CFBE41o^−^ cell surfaces treated with Ctl ASO or TMEM16a ASO (24 h, 50 nM) using a crystal violet assay. The dotted line indicates the maximum cell area, representing the surface of cells without bacteria (*n* = 5). Data are presented as mean ± SD with a 95% confidence interval. ANOVA followed by Dunnett’s and Bonferroni’s post hoc test. ∗∗*p* ≤ 0.01; ∗∗∗*p* ≤ 0.001; ∗∗∗∗*p* ≤ 0.0001.

On CF primary cells cultured in ALI with class I mutations (2184delA/W1282X) treated with TMEM16a ASO, we observed a strong and significant increase in the UTP response (*p* < 0.01) (Figures 2A and 2B). TMEM16a ASO also induced significant and robust activation of mucociliary clearance, as shown by the movement of fluorescent beads across all tested mutations (Figures 2C–2F). The effects of ETI on CFTR were significantly positive in cells with at least one F508del mutation (Figures 2D and 2E) but not in cells carrying two class I mutations (Figure 2F). Interestingly, the combination of TMEM16a ASO and ETI showed additive effects on F508del/F508del cells compared to cells treated with either TMEM16a ASO or ETI alone. However, a significant increase in Cl^−^ efflux was observed in cells treated with TMEM16a ASO (45.5 ± 0.3 vs. 96.3 ± 0.3) (Figure 2F).

In addition, TMEM16a ASO had no direct or intrinsic bacteriostatic or bactericidal effect on *Pseudomonas aeruginosa*, the most prevalent bacteria present in CF lungs (Figure 2G). Similar results were observed in the presence of cells (Figure 2H), indicating that the treatment was not involved in cellular defense (Figure 2I).

### TMEM16a ASO is specific and not toxic

**Figure 3:**
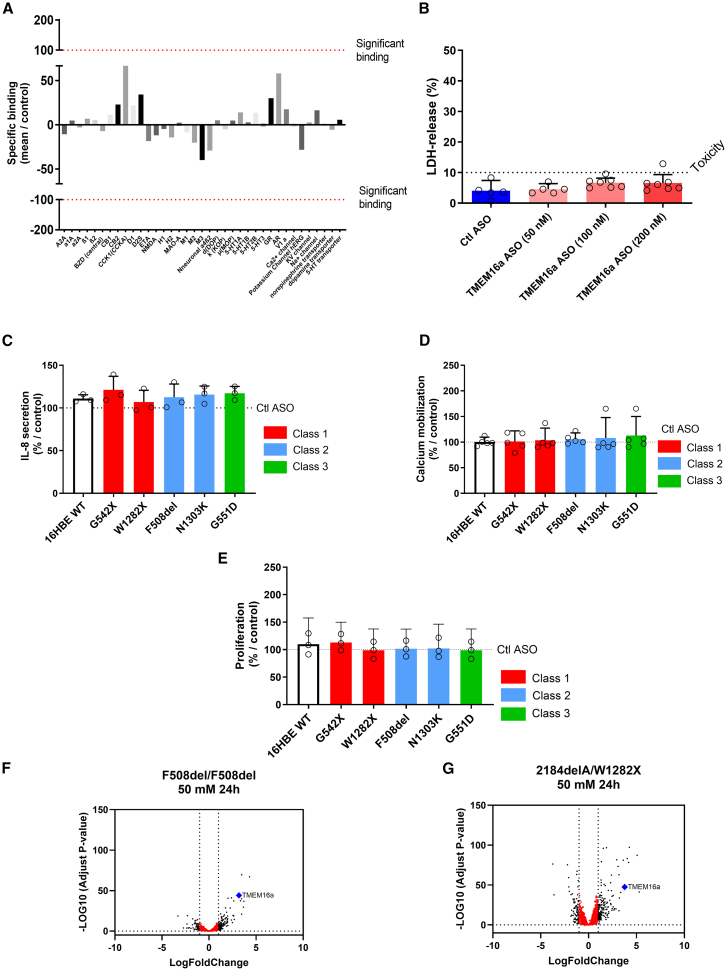
TMEM16a ASO is specific and not toxic (A) The results of the binding profile for TMEM16a ASO were expressed as a percentage of inhibition of control-specific binding, calculated as [(100 − measured specific binding/control-specific binding) × 100], obtained in the presence of TMEM16a ASO. Binding is deemed positive if it exceeds 100%. (B) Cytotoxicity was evaluated by quantifying lactate dehydrogenase (LDH) in CFBE41o^−^ treated with Ctl and TMEM16a ASO (*n* = 7). (C) The IL-8 secretion from the cells treated with TMEM16a ASO was measured using ELISA and normalized to the secretion from the cells treated with Ctl ASO (*n* = 3). (D) Mean peak values of ionomycin responses (10 μM) were assessed in 16HBE cell lines with various mutations labeled with fluo-4 after transfection with TMEM16a ASO or Ctl ASO (50 nM) for 24 h for each cell line (*n* = 5). (E) Proliferation was assessed in 16HBE cell lines with various mutations following TMEM16a ASO transfection or Ctl ASO transfection (50 nM) for 24 h (*n* = 3). (F and G) Volcano plot of transcriptomic analysis of RNA changes between hBEC cells cultured in ALI (*n* = 5 cultures per individual), treated with TMEM16a and Ctl ASO (50 nM, 1 h) for 3 days before mRNA extraction and after 24 h of incubation. (F) hBEC cells were obtained from a pwCF with a class II mutation (F508del/F508del), and (G) was sourced from a pwCF with class I mutations (2184delA/W1282X). Data are presented as mean ± SD with a 95% confidence interval. ANOVA followed by Dunnett’s and Bonferroni’s post hoc test. ANOVA with Dunnett’s and Bonferroni’s post hoc tests found no significant results.

We conducted an *in vitro* pharmaceutical profiling as described by Bowes et al. to identify undesirable off-target interactions.^19^ No notable adverse drug binding of TMEM16a ASO was observed (Figure 3A; Table 1). *In vitro* pharmacological profiling involves screening compounds against various targets (receptors, ion channels, enzymes, and transporters) that differ from the intended therapeutic target(s) to identify specific molecular interactions that could lead to adverse human drug reactions. We also evaluated TMEM16a ASO toxicity after 24 h of treatment on CFBE41o^−^ cells by measuring lactate dehydrogenase (LDH) levels and identified no differences up to 200 mM (Figure 3B). Similar results were obtained on hBEC (data not shown). No significant induction of cytokines, including interleukin-8 (IL-8), the most activated cytokine in CF lungs, was observed in cell lines with different mutations (Figures 3C and S1A). Our study also demonstrated that ASO TMEM16a does not activate TMEM16a by increasing intracellular Ca^2+^ (Figure 3D). Similarly, we did not observe a significant change in cell proliferation compared to cells treated with the Ctl ASO (Figure 3E).

**Table 1:** Top 10 mRNA fold changes observed after transcriptomic analysis of CF human primary cells cultured in ALI treated with TMEM16a ASO

F508del/F508del			2184delA/1282X		
mRNA	Log fold change	−Log10 *p* value	mRNA	Log fold change	−Log10 *p* value
SPDEF	2.6	37.3	SLC26A4	3.2	17.8
CDH6	2.7	14.4	ALOX15	3.2	33.8
TMEM16a	3.2	44.1	C1QTNF1	3.4	40.8
DPP4	3.3	37.4	DPP4	3.6	81.2
SLC26A4	3.5	20.9	TMEM16a	3.8	47.6
SERPINB4	3.5	39.9	SERPINB4	4.0	83.3
CDH26	3.5	69.5	SERPINB2	4.2	78.3
ALOX15	3.7	29.6	CDH26	4.3	97.3
SERPINB2	3.7	36.8	SERPINB10	5.1	87.3
SERPINB10	4.3	67.0	NOS2	5.3	41.3

Cultured during 24 h (50 nM), including the log fold change and −log10 p value, in a CF individual with F508del/F508del mutations (left 3 columns) and in a CF individual with 2184delA/1282X mutations (right 3 columns).

To confirm the specificity of the ASO TMEM16a, we conducted a transcriptomic analysis on primary cells cultured in an air-liquid interface with a class II mutation (F508del/F508del) and a class I mutation (2184delA/W1282X). Cells were treated with 50 nM for 24 h (Figures 3F and 3G), 48 h (Figures S1B and S1C), and 200 nM (data not shown). TMEM16a mRNA is overexpressed in all cases and ranks in the top 10 genes when cells are treated with TMEM16a ASO compared to the Ctl ASO (Table 1, left and right), with a log fold change between 3.2 and 3.8. Consistent with the results above, our transcriptomic analysis showed no significant increase in RNAs associated with inflammation or evidence of cellular toxicity, even at a concentration of 200 nM for 48 h.

### Subcutaneous administration allows for optimal distribution and is not toxic to mice

**Figure 4:**
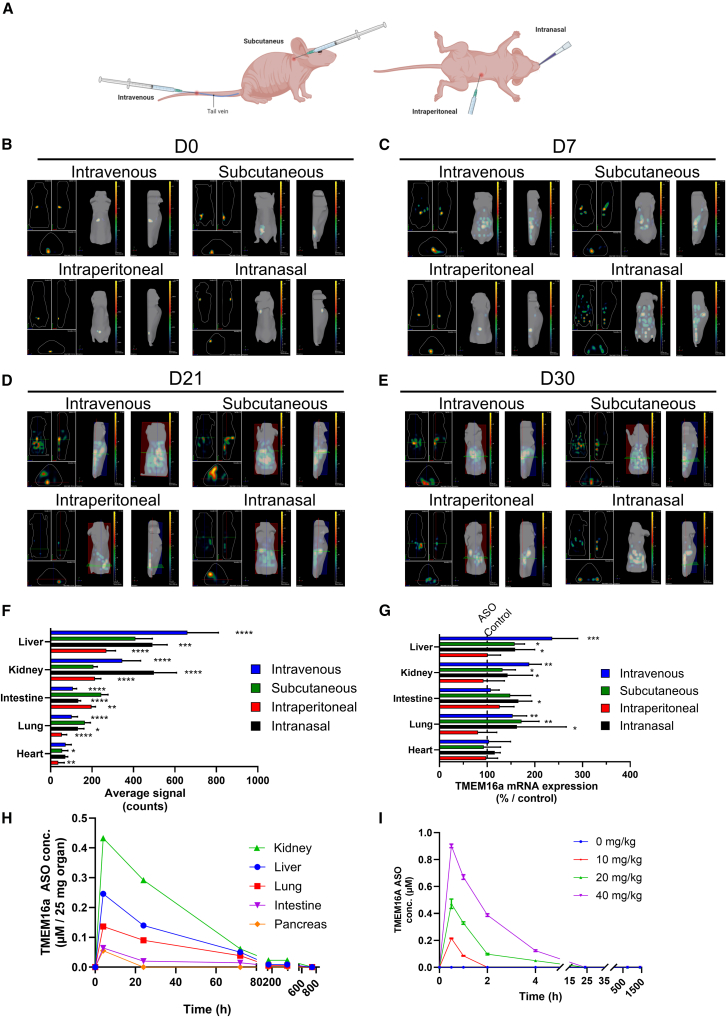
Subcutaneous injection induces optimal ASO biodistribution, which is sustained and does not induce toxicity (A) Diagram illustrating various routes of administration in mice. (B–E) *In vivo* imaging of nude mice following intravenous, subcutaneous, or intraperitoneal injections or intranasal instillation of fluorescent TMEM16a ASO (10 mg/kg) at various time points: (B) day 0 (D0), (C) day 7 (D7), (D) day 21 (D21), and (E) day 30 (D30) (*n* = 3/group). (F) Analysis of fluorescence intensity in organs from mice sacrificed 30 days after treatment (*n* = 5/group). (G) Quantification of TMEM16a expression in organs 30 days after the injection of the TMEM16a ASO compared to naive mice (*n* = 5/group). (H) Quantification of TMEM16a ASO concentration at different times (4 h, 24 h, 3 days, 1 week, 2 weeks, and 4 weeks) in 25 mg kidney, liver, lung, intestine, and pancreas (*n* = 3/group). (I) Quantification of plasma TMEM16a ASO concentration at different times (30 min, 1 h, 2 h, 4 h, 24 h, 800 h) (*n* = 3/group). Data are presented as mean ± SD with a 95% confidence interval. ANOVA followed by Dunnett’s and Bonferroni’s post hoc test. ANOVA with Dunnett’s and Bonferroni’s post hoc tests. ∗*p* ≤ 0.05; ∗∗*p* ≤ 0.01; ∗∗∗*p* ≤ 0.001; ∗∗∗∗*p* ≤ 0.0001.

To determine the optimal route of administration, fluorescent TMEM16a ASO was administered to nude mice (10 mg/kg) through intravenous, subcutaneous, or intraperitoneal injections or intranasal instillation (Figure 4A). Fluorescence in mice was recorded weekly for 1 month (Figures 4B–4E). With the intraperitoneal injection, the drug only dispersed to a limited extent, even 30 days after administration. In contrast, intravenous, subcutaneous, and intranasal routes showed better distribution in all tissues as early as 7 days (Figures 4C and 4D). After day 21, we observed a decline in fluorescence levels for all conditions (Figure 4E). At 30 days, the mice were euthanized, and the fluorescence of TMEM16a ASO in the major organs was quantified (Figure 4F). For all routes of administration, as expected for other studies with ASOs, we observed a significant accumulation in the kidney and liver. A more effective distribution of fluorescence is noted with intranasal or subcutaneous administration (Figure 4F). On day 30, we observed a significant increase in TMEM16a mRNA expression in the lung after intravenous, intranasal, and subcutaneous administration compared to the Ctl ASO (Figure 4G). The same results were found in the liver, kidney, and intestine. Pharmacokinetic and pharmacodynamic experiments using mass spectrometry were performed. Mice were administered TMEM16a ASO subcutaneously (10 mg/kg), and we demonstrated rapid diffusion of the ASO to the organs. We observed a peak at 4 h, followed by a gradual decrease in the presence of ASO over time. We detected the presence of TMEM16 ASO at a very low concentration on day 30 in various organs (Figure 4H). Since the primary target organs in humans are the lungs and, in mice, the intestines, we have chosen subcutaneous injection, a common method of ASO administration. In plasma, TMEM16a ASO was undetectable after 24 h (Figure 4I). Toxicity in mice was evaluated *in vivo* by observing general signs (morbidity and mortality, clinical symptoms, body weight, food, and water intake) three times per week for 14 days. Mice were injected subcutaneously with six doses ranging from 0 to 500 mg/kg. At 50 times the active dose (500 mg/kg), we observed no significant differences in the mice’s behavior, macroscopic autopsy, or histological analysis (data not shown). We completed this study by analyzing the tissue after 1 year of treatment. A portion of the tissue was embedded in paraffin, and the remainder was stored at −80°C for subsequent examination of myeloperoxidase (MPO). Sections 4 μm thick were stained with hematoxylin and eosin before histological examination (Figures S2A and S2B). Tissue injuries were scored according to the following criteria: hemorrhage (+1), cell infiltration (+1), and remodeling (+1). The data were then compared to those of the wild-type (WT) mice tissues. After 1 year of treatment, we observed no significant differences in the mice’s behavior, macroscopic autopsy, histological analysis (Figure S2C), or in MPO quantitation that reflected neutrophil infiltration (Figure S2D).

In blood samples, parameters reflecting liver toxicity (ALT and AST), kidney toxicity (urea), and pancreas toxicity (amylase) remained consistent across all tested doses (Figures S3A–S3D), and no differences were found in the hematological analysis (data not shown). In CF mice receiving repeated injections every 15 days for over 200 days, the levels of transaminases, urea, and creatinine (Figures S3E and S3F) were comparable to those in control mice. The histopathological observations of the liver, kidney, intestine, and lung were also similar (data not shown).

### TMEM16a ASO decreases the physiopathology of CF mice

**Figure 5:**
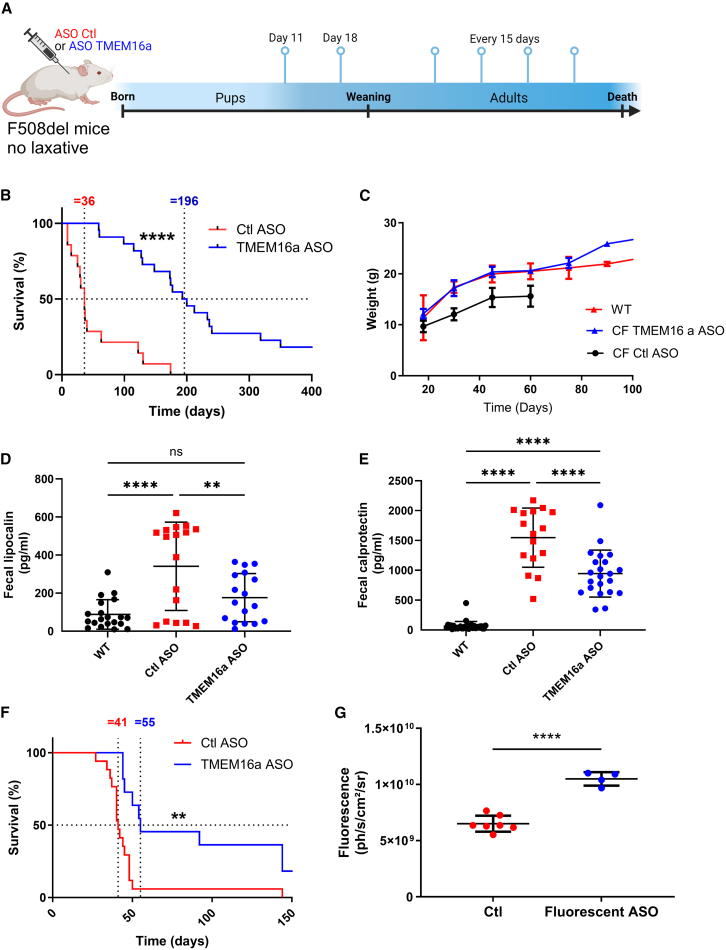
TMEM16a ASO treatment enhances viability and weight gain, while reducing intestinal inflammation in F508del mice (A) TMEM16a ASO and Ctl ASO (10 mg/kg) were injected subcutaneously on days 11 and 18 and then every 15 days thereafter into 129-cftrtm1Eur CF mice (F508del/F508del). (B) Kaplan-Meier survival curves for overall survival analysis in CF mice treated with either Ctl ASO or TMEM16a ASO. The data represent the average of more than 100 mice. (C) The weight values presented are averages based on as many as 24 mice per group. The values represent the mean and 1 SD. Ages vary by ±3 days. (D and E) ELISA analyzed fecal lipocalin (D) and calprotectin (E) (*n* = 20/group). (F) Kaplan-Meier survival curves for the overall survival analysis in CF pups born to parents treated with TMEM16a ASO or Ctl ASO. Data represent the mean of at least 20 mice per group. The dotted lines indicate median survival when the staircase survival curve intersects 50%. (G) Quantification of fluorescence in the pups after birth. The gestating females were treated with fluorescent ASO TMEM16a or vehicle (10 mg/kg) 7 days before giving birth (*n* = 7/group). Survival was evaluated between groups using the Kaplan-Meier survival analysis and log rank test for (B) and (F) and ANOVA, followed by Dunnett’s, Bonferroni’s, and Tukey’s post hoc tests (D and E). ns, not significant; ∗∗*p* ≤ 0.01; ∗∗∗*p* ≤ 0.001; ∗∗∗∗*p* ≤ 0.0001.

**Figure 6:**
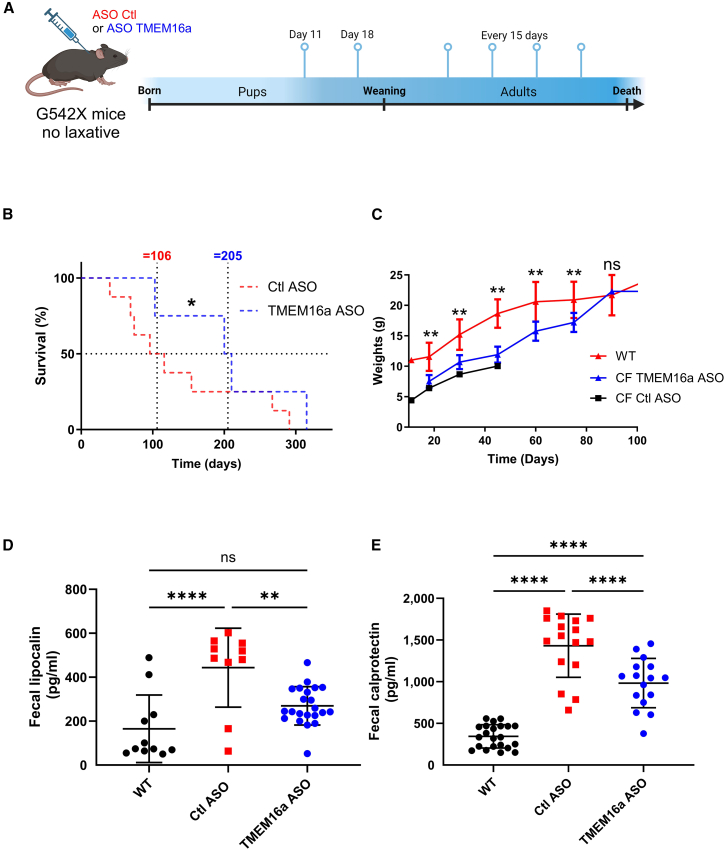
Treatment with TMEM16a ASO increases viability and weight gain and decreases intestinal inflammation in G542X mice (A) TMEM16a ASO and Ctl ASO (10 mg/kg) were subcutaneously injected on days 11 and 18 and subsequently every 15 days into Cftrem3Cwt CF mice (G542X/G542X). (B) Kaplan-Meier survival curves for overall survival analysis in CF mice treated with Ctl ASO or TMEM16a ASO show data as the mean of more than 25 mice. (C) The histogram of the weight values presented represents averages based on up to 24 mice per group. These values represent the mean and 1 SD. Ages are ±3 days. (D and E) Fecal lipocalin (D) and calprotectin (E) were analyzed by ELISA (*n* ≥ 11). Survival was evaluated between groups using the Kaplan-Meier survival analysis and log rank test (B) and ANOVA, followed by Dunnett’s and Bonferroni’s post hoc tests (C–E). ns, not significant; ∗∗*p* ≤ 0.01; ∗∗∗*p* ≤ 0.001; ∗∗∗∗*p* ≤ 0.0001.

In the following experiments, we used CF F508del mice (Figure 5) and CF G542X mice (Figure 6). We treated CF mice before weaning, on days 11 and 18 after birth, with subcutaneous injections of Ctl ASO or TMEM16a ASO (10 mg/kg). We continued treatment every 15 days after weaning, as illustrated in Figures 5A and 6A. With ASO TMEM16a treatment, median survival ranged from 36 days in control mice to 196 days in F508del mice treated with TMEM16a ASO (*p* < 0.0001) (Figure 5B). As previously reported, CF mice died from severe intestinal obstructions, especially in the ileum. Therefore, after only two injections on days 11 and 18, the treatment appeared to provide temporary protection to the F508del CF mice (Figure S3G). In F508del mice, there was no significant difference in weight gain compared to WT (+/+) mice and CF mice treated with TMEM16a ASO (Figure 5C). Comparing these results to those of the control mice is challenging due to the high mortality rate in younger mice and the small number of mice surviving after 60 days or more (Figure 5B). Macroscopic observation reveals no difference in the intestines of CF treated adult mice compared to those of normal mice (data not shown). In contrast, untreated mice or those treated with Ctl ASO exhibit significant intestinal occlusion, as previously described (data not shown). After at least 4 weeks of treatment, we also assessed gut inflammation in mice using two fecal biomarkers—lipocalin (Figure 5D) and calprotectin (Figure 5E)—which are secreted by neutrophils and monocytes, respectively. We observed notably higher fecal lipocalin levels in F508del CF mice. Following treatment with TMEM16a ASO, we observed a significant decrease in values compared to the value obtained (*p* < 0.01) (Figure 5D), returning to levels seen in WT mice. The same results were obtained with fecal calprotectin; however, the difference between WT and TMEM16a ASO mice remained significant (Figure 5E). In CF treated mice, less ileal obstruction with more fluid feces was observed compared to non-treated mice (data not shown).

As previously reported, macroscopic analysis of the vas deferens showed that male −/− mice treated with Ctl ASO displayed numerous malformations with significant variation.^20^^,^^21^^,^^22^ In most cases, we observed a 30% unilateral or bilateral shortening of the vas deferens in mice treated with the Ctl ASO, compared to 6.5% in TMEM16 ASO mice. In 79% of cases, the vas deferens narrowed at specific locations, a phenomenon not observed in WT mice (Table 2). These observations may elucidate the vas deferens atresia in these mice, rendering them non-functional and leading to infertility (Table 2; Figure S4A, second generation, left). Most TMEM16a ASO-treated male mice were able to reproduce during the study, unlike the majority of mice treated with Ctl ASO.

**Table 2:** Vas deferens abnormalities observed in F508del mice

	+/+ mice (*n* = 30)	Ctl ASO −/− mice (*n* = 24)	TMEM16a ASO −/− mice (*n* = 32)
Length, %	100	−31.5	−6.25
Atresia, %	9	79.10	18.75
Absence, %	0	16.60	0
Fertility, %	96.67	4.16	88.23

In our study, homozygous mice (F508del/F508del) chronically treated on day 11 after birth (Figure S4A, second generation, right) were able to reproduce, and the pups were viable (Figure S4A; third generation, Figure S4B and S4C). These third-generation CF mice, born to parents treated with TMEM16a ASO, survived the weaning stage and had a longer lifespan than CF mice born to untreated parents, even though these third-generation mice received ASO Ctl treatment (control mice median survival: 55 vs. 41) (Figure 5F).

We monitored fluorescence in newborn pups by injecting fluorescent TMEM16a ASO into gestating female mice before delivery. We observed a significantly higher level of fluorescence (Figure 5G), confirming ASO transmission from the pregnant female to the pups. Additionally, we observed fewer abnormalities in the male reproductive systems of mice treated with TMEM16a ASO (Figures S4D and S4E). The results for G542X mice (Figure 6) were similar to those for F508del mice (Figure 5), showing significance and an increase in life expectancy from 106 to 205 days (*p* < 0.0001) (Figure 6B). Regarding weight gain, no significant difference was observed in G542X mice treated with TMEM16a ASO until day 90 as compared to WT mice (Figure 6C). In this CF model, we observed significantly higher levels of fecal lipocalin and fecal calprotectin than in WT mice (Figures 6D and 6E).

## Discussion

Although new CF modulator treatments work well for many individuals with CF, they are only suitable for a subset of patients, leaving others with unresolved medical issues.^2^ This study provides data supporting a new therapeutic approach using an ASO that targets the alternative chloride channel TMEM16a, aiming to restore its expression and indirectly compensate for the deficiency of CFTR protein in CF). This new approach is independent of disease-causing CFTR mutations and could benefit all pwCF, particularly those who are currently ineligible for curative treatments. The concept of rectifying the ion transport defect regardless of the *CFTR* genotype emerged in the early 1990s, when studies showed that triphosphate nucleotides such as adenosine-5′-triphosphate and UTP activate Cl^−^ efflux in both normal and CF airway epithelia.^23^ Before the identification of airway epithelial CaCC in 2008, denufosol was developed to treat CF lung disease. Despite some promising results in pwCF, the second phase 3 trial failed to demonstrate any benefits due to a lack of specificity and low stability in biological fluids.^24^ This approach was later abandoned due to limited understanding of the involved channels. Our approach, utilizing ASOs in CF, an innovative therapeutic tool that binds specifically to the targeted mRNA through complementary Watson-Crick base pairing, can prevent such issues, as ASOs do not disrupt calcium (Ca^2+^) mobilization. This study also reveals that activating the TMEM16a channel enhances Cl^−^ activity and improves mucociliary clearance *in vitro* by modifying ionic movement in the airways, while mucin expression remains unchanged. In our approach, we have treated the mice before the onset of the disease to prevent their death. Based on the experience already acquired with CFTR modulators in pwCF and with antisense oligonucleotides targeting ENaC in mice, we can assume that treatment in humans, once the disease is already established, could have beneficial effects.^25^

Our research shows that TMEM16a increases the survival rate of CF mice following intestinal obstruction at an early age. It also relieves other symptoms, including infertility and intestinal blockage. The reduced expression of these channels in the gut contributes to the observed pathology in CF mice. Notably, the improvements in other organs are intriguing, as they have not been documented in pwCF treated with Trikafta. CF mouse models do not permit the observation of respiratory effects because pulmonary involvement is minimal in CF mice due to the presence of TMEM16a channels that are already present in the airways.^26^^,^^27^ It would be valuable to investigate the effects of treatment on other CF animal models that are more relevant to pulmonary desease, such as ferrets and pigs.^28^ CFTR knockout (KO) pigs are significantly affected by intestinal disease. All newborn piglets experience meconium ileus, which is fatal unless corrective surgery is performed soon after birth to relieve the obstruction. It may be worth treating the mother to see whether there is any improvement in the newborns. Phe508del pigs are also impacted, although the intestinal phenotype is somewhat less severe. New CF ferret models have been generated, including a gut-corrected CF KO ferret, which exhibits multi-organ disease and responds to the CFTR potentiator ivacaftor.^29^ However, there are currently few data available on TMEM16a expression in the airways of these models. These limitations indicate that using this model is complicated and should only be considered after a more thorough characterization of TMEM16a. Although these models have specific limitations, models representing class I mutations are still unavailable.

A major challenge of this strategy is to maximize the efficacy and minimize the toxicity of ASO drugs. This article presents various results from studies on cells and mice, demonstrating the low toxicity of this approach, even after extended treatment. This confirms other strategies using ASO in CF models, where ASO is safe and well tolerated.^30^^,^^31^ The strategies used for patients are either tailored to specific CFTR mutations or target other ion channels, such as the hyperactivated ENaC channel in pwCF.^32^ ASO technology has emerged as an appealing therapeutic modality for CF; however, this field has largely remained unexplored in the context of cellular studies.^33^ The potency and toxicity of ASOs depend on their chemical modifications, off-target effects, and delivery methods.^32^ In our experiments, we did not observe off-target effects on the different CF models analyzed. The transcriptomic analysis results showed indirect connections between this channel and many proteins. This indirect connection may arise from protein and channel interactions, but it could also be attributed to increased chloride activity. Regarding the transcriptomic analysis, TMEM16a ranks among the top 10 most highly expressed and significant genes across all tested conditions. We also observed an increase in other genes, which may be an indirect consequence of the expression or activity of TMEM16a, as previously demonstrated for the CFTR protein.^34^^,^^35^^,^^36^

Our research shows that TMEM16a ASO has strong specificity, minimal toxicity, and good stability *in vivo*. An important aspect of oligonucleotide use is the route of administration envisaged and its accumulation in specific tissues, such as the liver and kidneys. Studies have shown that pulmonary administration and intravenous injection result in the same accumulation of oligonucleotides in the liver of mice 7 days after administration.^37^ In our study, administering every 15 days is enough to improve the animals’ survival significantly. These data are consistent with the literature, and the results obtained from the persistence of TMEM16a ASO from females to pups may explain the increase in pup survival observed in females treated only with Ctl ASO.^38^ Under this condition, chronic administration of TMEM16a ASO twice per month has increased the survival of F508del mice from 36 to 196 days, and from 106 to 205 days for G542X mice. This strategy minimizes adverse side effects by using a minimal dose to restore TMEM16a activity without causing any negative effects from over-induction.^16^ Through this approach, we have not observed any modifications in the expression or activity of other chloride channels. Concerns have been raised regarding whether the activation of TMEM16a could negatively impact mucus secretion or cause vasoconstriction in the bronchi, as noted in another approach.^39^ Our *in vivo* models showed no adverse effects, even when the animals were treated for extended periods (over 1 year). The mucus secretion induced by TMEM16a overexpression is more complex, underscoring the limitations of our understanding of cellular lung health physiology.^40^ A publication by Amaral’s team offers a partial answer by showing that TMEM16a does not drive mucus production, which is confirmed by observation in the airways of CF mice.^41^ This strategy is also open for discussion, as many articles indicate that TMEM16a is present in numerous cancers due to its pro-proliferative effects.^42^^,^^43^ Our objective was not to overexpress and drastically increase TMEM16a activity but rather to selectively restore the profile seen in non-CF cells in a targeted way to avoid adverse effects. Furthermore, no tumors were observed in CF mice that were repeatedly treated with TMEM16a over 1 year.

Interestingly, the effects of TMEM16a ASO were not limited to lung improvements but could also affect other CF manifestations when administered subcutaneously, as demonstrated by the effects observed on gastrointestinal obstruction in our CF mice. Gastrointestinal symptoms were identified as the second priority through a questionnaire completed by over 1,100 pwCF,^44^ even though some abdominal symptoms appear to be reduced with ETI therapy.^44^ Another unexpected result was the improvement in fertility of male CF mice by restoring vas deferens abnormalities when the mice were treated very early.^20^^,^^21^^,^^22^ These symptoms in mice are similar to those observed in CF sufferers, although they differ during the development of the vas deferens.^1^ In humans, 98% of men with CF are infertile due to obstructive causes, particularly congenital bilateral absence of the vas deferens, with abnormalities at birth.^45^ In mice, vas deferens development continues after birth, so the positive effects observed with TMEM16a ASO are possible and could explain the results; however, these results are not directly applicable to humans.^46^ The observations noted will require targeted additional studies, such as histological analyses during vas deferens development and functional assessments of spermatozoa. Furthermore, these findings need further examination to clarify the potential role of TMEM16a in vas deferens formation, since the available data are very limited.^47^^,^^48^^,^^49^ Nevertheless, these data indicate that this systemic treatment could benefit other organs, such as alleviating intestinal symptoms in pwCF. Unfortunately, mice do not respond to Trikafta, making comparisons with this model impossible. This article demonstrates that TMEM16a produces effects similar to Trikafta in cells with class II mutations. Additionally, we have shown that our strategy can also benefit models with class I mutations, where Trikafta falls short. Moreover, our approach utilizing oligonucleotides is highly complementary to the Trikafta strategy, as the differing mechanisms involved allow for enhanced effectiveness. In contrast to numerous CF strategies, this study proved effective in class I and II carrier cells and mice. Like ours, one group proposed a more classical approach utilizing the molecule ETX001, which recently has been developed to target TMEM16a. As observed in our approach, ETX001 operates independent of Ca^2+^ efflux, ensuring specificity and minimizing secondary effects.^50^ Similarly, as previously demonstrated, this strategy restored Cl^−^ efflux both *in vitro* and *in vivo* in an ovine model.^51^ While the two approaches have similar goals, the TMEM16a ASO acts as a corrector, specifically a facilitator of TMEM16a expression.^16^ In contrast, ETX0001 functions primarily as a potentiator, requiring TMEM16a expression in this context. Nonetheless, the proposed approach, which utilizes ETX001 and our TMEM16a method, could work together to activate the TMEM16a channel. This approach could be very beneficial for patients who are non-responders to modulators but require further analysis.

In conclusion, targeting TMEM16a can address multiple symptoms through a CFTR-independent strategy, applicable to all pwCF alone or in combination with other CFTR-targeted approaches for eligible patients.

## Materials and methods

### Antisense oligonucleotides

TMEM16a ASO and Ctl ASO were synthesized by Qiagen (France) as sodium salts. The purification was performed using high-performance liquid chromatography (HPLC) and sodium exchange, and the resulting material was provided for each study as lyophilized powder. Physiological serum was used for dilution of TMEM16a ASO and Ctl ASO. ASO was modified by adding phosphorothioate oligonucleotide linkages and LNA. TMEM16a ASO and Ctl ASO are composed of 15 nt. TMEM16a ASO was designed to specifically bind the 3′ UTR of TMEM16a in both human and mouse sequences. The sequence of TMEM16a ASO used was AATCTTTGGTAGTAA, and the Ctl ASO sequence was ACGTCTATACGCCA.

### Cell culture and transfection

As previously described, CFBE41o^−^, 16HBE14o^−^, CUFI-5 (LGC, France), and CFPAC-1 cell lines were cultured and transfected with ASO (Ctl or TMEM16a).^51^^,^^52^ The 16HBE14o^−^ cell lines with different *CFTR* mutations were generated by the Cystic Fibrosis Foundation.^53^ Primary hBECs were isolated from bronchial biopsies and supplied by Epithelix SARL (Switzerland).^16^ Following the manufacturer’s instructions, proliferation was quantified using the CyQUANT NF cell proliferation assay (Thermo Fisher Scientific, France).

### Bacterial strains and cultures

An inoculum of luminescent PAO1 strain of *P. aeruginosa* was grown in Luria-Bertani medium at 37°C. The bacterial growth of *P. aeruginosa* was monitored over time by measuring luminescence with a plate reader. The multiplicities of infection (MOIs) used were 0.01, 0.1, 1, and 10.

### TMEM16a chloride channel activity assay

Short-circuit current (Isc) was measured under voltage clamp (Physiologic Instruments, USA). The differential composition of basal and apical Ringer’s solutions created the chloride gradient across the epithelium. The basal Ringer’s solution contained 145 mM NaCl, 3.3 mM K_2_HPO_4_, 10 mM HEPES, 10 mM d-glucose, 1.2 mM MgCl_2_, and 1.2 mM CaCl_2_. The apical solution contained 145 mM Na-gluconate, 3.3 mM K_2_HPO_4_, 10 mM HEPES, 10 mM d-glucose, 1.2 mM MgCl_2_, and 1.2 mM CaCl_2_. Inhibitors and activators were added after stabilization of baseline Isc: sodium channel blocker amiloride (100 mM), forskolin to induce cAMP activation of CFTR (10 μM), CFTR inhibitor CFTR-Inh172 (10 μM) to specifically inhibit CFTR, UTP (100 μM) to challenge the purinergic calcium-dependent Cl^−^ secretion, and Ani9 (10 μM) was utilized as a specific inhibitor of TMEM16a. The short-circuit currents (Isc) were measured at 1 measurement per 5–20 s. The change of Isc upon CFTRInh172 application served as an index of CFTR function. Chemicals were purchased from Sigma (USA).

In some experiments, TMEM16a channel activity was assessed by iodine quenching of the halide-sensitive YFP-H148Q/I152L protein.^54^

### Mucociliary clearance assay

As previously described, hBECs were transfected with Ctl ASO or TMEM16a ASO daily for 1 h over 3 days and/or stimulated with ETI (VX-445, elexacaftor at 3 μM; VX-661, tezacaftor at 10 μM; and VX-770, ivacaftor at 1 μM; Euromedex, France) for 24 h.^16^

### *In vitro* pharmaceutical profiling

Pharmaceutical profiling was determined *in vitro*, following the recommendation of Bowes et al.^20^ Eurofins Cerep (France) performed all experimental studies.

### Cytotoxicity evaluation

The LDH cytotoxicity assay was conducted *in vitro* using the LDH-based CytoTox 96 non-radioactive cytotoxicity assay, following the manufacturer’s guidelines (Promega, France).

### Calcium mobilization

After treatment with TMEM16a ASO, cells were loaded with Fluo-4 dye, as previously described.^55^ The cells were incubated with the dye at 37°C for 30 min, washed with calcium-free PBS, and then activated with 10 μM ionomycin. The cells were subsequently recorded using a plate reader (BMG LabTech, France).

### Transcriptomic experiments

For cells, miRNA and RNA were extracted using a Nucleospin miRNA kit (Macherey-Nagel, Germany). The concentration and quality of RNA and miRNA were evaluated using a NanoDrop spectrophotometer, and library quality and size range were assessed using a Bioanalyzer (Agilent Technologies, Belgium). The library and transcriptomic analysis were performed at the VIB Nucleomics Core (Belgium). Samples were indexed to allow for multiplexing. Libraries (2 nM) were sequenced on an Illumina HiSeq 4000 platform (Illumina, USA). Single-end reads of 50-bp length were produced with a minimum of 1 million reads per sample. The alignment files were generated for the reference genome using STAR version 2.5.2b. Reads from the alignment that are non-primary mappings or have a mapping quality of <20 were removed using Samtools version 1.15.1. Quality control of raw reads was performed with FastQC version 0.11.7. The reads were sorted and indexed according to the chromosomes, and the resulting BAM files were indexed with Samtools. The main sources of sample-specific variation (library size and RNA composition) were corrected using full quantile normalization with the EDASeq package from Bioconductor. For transcriptomic analysis and to correct for the potential batch effect, CombatSeq from the sva-devel R package (https://doi.org/10.1093/nargab/lqaa078) was used.

Independent replicates were incorporated, and validation was conducted using quantitative PCR to confirm mRNA expression. We validated both significant (TMEM16a, SLC26A4, ERPINB4) and nonsignificant (CXCL8, IL-6, SLC26A14, CFTR, and ENaC) mRNA.

### CF mouse model

BALB/cAnNRj-Foxn1 nu/nu were purchased (Janvier-Labs, France). The 129-Cftrtm1Eur model mice homozygous for the F508del mutation and their wild-type littermates were obtained from CDTA-CNRS (France). Cftr^em3Cwt^ G542x model mice homozygous for the G542X mutation and their WT littermates were provided by Dr. Craig A. Hodges (Case Western Reserve University, Cleveland, OH). During the TMEM16a ASO treatment, no laxative was provided as previously described to avoid interfering with the treatment. Studies were approved by the Institutional Animal Care and Use Committee (APAFIS no. 38427-2022091211033928 v8 and APAFIS no. 43883-2023062011193610 v7).

### TMEM16 ASO quantification

The analyses were performed by HPLC coupled with mass spectrometry (MS). Liquid chromatography was performed on the Ultimate 3000 series Pump System (Thermo Scientific). Detection was carried out on a mass spectrometer, which included an electrospray ionization source and an ion trap mass analyzer (Bruker), as well as an ultraviolet-visible spectroscopy detector (VWD3100, Thermo Scientific). Raw data were acquired and processed using Hystar 3.2 and DataAnalysis/QuantAnalysis (Bruker), respectively. The linearity of the calibration curve was tested on TMEM16 ASO with HPLC-MS/MS analysis. Frozen tissue samples (organs) were homogenized with the GentleMACS dissociator device using 250 mg tissue per 1,000 μL ATL Buffer and lysed overnight with Proteinase K.

### Toxicity assessment

C-Ris Pharma (France) conducted the *in vivo* toxicity protocol on healthy CD-1 mice. Histology and blood analysis were carried out on mice treated at 500 mg/kg (50 times the effective dose) and sacrificed 14 days after subcutaneous injection. After sacrifice, the organs were collected, embedded in paraffin, and stained with hematoxylin and eosin before being sliced and analyzed.

### Non-invasive fluorescence assessment *in vivo*

Nude mice (BALB/cAnNRj-Foxn1 nu/nu) were treated with fluorescent TMEM16a ASO (Eurogentec, Belgium; excitation wavelength 640 nm/emission wavelength 680 nm; 10 mg/kg) through various routes of administration to avoid autofluorescence in the mice’s coats. Imaging was performed using the IVIS Spectrum imaging system (PerkinElmer, France) through two-dimensional epifluorescence imaging at specified times after probe injection to optimize readouts. Three-dimensional images were then generated using IVIS software. After 30 days, the organs were extracted and isolated, and imaging was conducted using the IVIS Spectrum imaging system. Fluorescence was recorded, and the images were analyzed and quantified using ImageJ version 1.54 software.

### Statistical analysis

Statistical analysis was performed as indicated in the respective figure legends, using GraphPad Prism 10.5.3 software (GraphPad Software, USA). Extracted contrasts included comparisons between different groups and pairwise comparisons of points in time within each condition. Raw *p* values were adjusted for multiple testing using the Benjamini-Hochberg procedure and genes with an adjusted *p* value. All figures show means and 95% confidence intervals.

## Data availability

The datasets generated and/or analyzed during this study are available in the supplemental information or from the corresponding author upon reasonable request.

## Acknowledgments

F508del mice were generated by the CF EMCs (Scholte et al.) and the sponsorship of the EUROCARECF (6^th^ framework coordination action program LSHM-CT-2005-018932). G542X mice were generated by the CF Mouse Resource Center at Case Western Reserve University, USA (Dr. Craig A. Hodges). The authors thank the Association Les Motards du Viaduc de Millau, France, for funding the project. The following french grants supported this work: “Blanche pour Vaincre la Mucoviscidose” (RF20220503020), “10.13039/501100006342Vaincre la Mucoviscidose” (RF20190502484, RF20200502752, and RF20180502589); SATT Lutech; Cystem Flow; 10.13039/100014865Fondation Maladies Rares (FSMR-070805); Anoat Therapeutics (collaboration agreement 21118A20); INSERM Transfert (MAT-PI-15170-A-03).

## Author contributions

O.T. conceived the project, designed the research, and supervised the study. C.M., N.R., M.D.W., T.F., G.D., M.L., V.G., F.S., and O.T. conducted the research and analyzed the data. O.T. and C.M. secured the funding and wrote the paper. All authors provided feedback and contributed to editing the manuscript.

## Declaration of interests

C.M., F.S., H.C., and O.T. are co-founders and board members of Anoat Therapeutics. TMEM16a ASO is patent protected. C.M., F.S., H.C., and O.T. are listed as co-inventors on the following patents: EP16736045.2, US15/739,366, EP18 783 539.2, US16/756,917, and EP22305163.2.

## Supplemental information

Document S1. Figures S1–S4

Document S2. Article plus supplemental information
