# Supplementary material for: Restoring chloride efflux in cystic fibrosis with TMEM16a antisense oligonucleotides
Source: Mol Ther. 2025 Sep 8;33(12):6463–78. doi: 10.1016/j.ymthe.2025.08.045 (PMC12703169; doi:10.1016/j.ymthe.2025.08.045)
Supplement: Document S1. Figures S1–S4 [file mmc1.pdf]

YMTHE, Volume *33*

Supplemental Information

Restoring chloride efflux in cystic fibrosis

with TMEM16a antisense oligonucleotides

Christie Mitri, Nathalie Rousselet, Pauline Bardin, Madara Dias Wickramanayaka, Tobias Foussignière, Gabrielle Dupuis, Marion Leblanc, Victoire Gournet, Florence Sonneville, Harriet Corvol, and Olivier Tabary

SUPPLEMENTAL INFORMATION

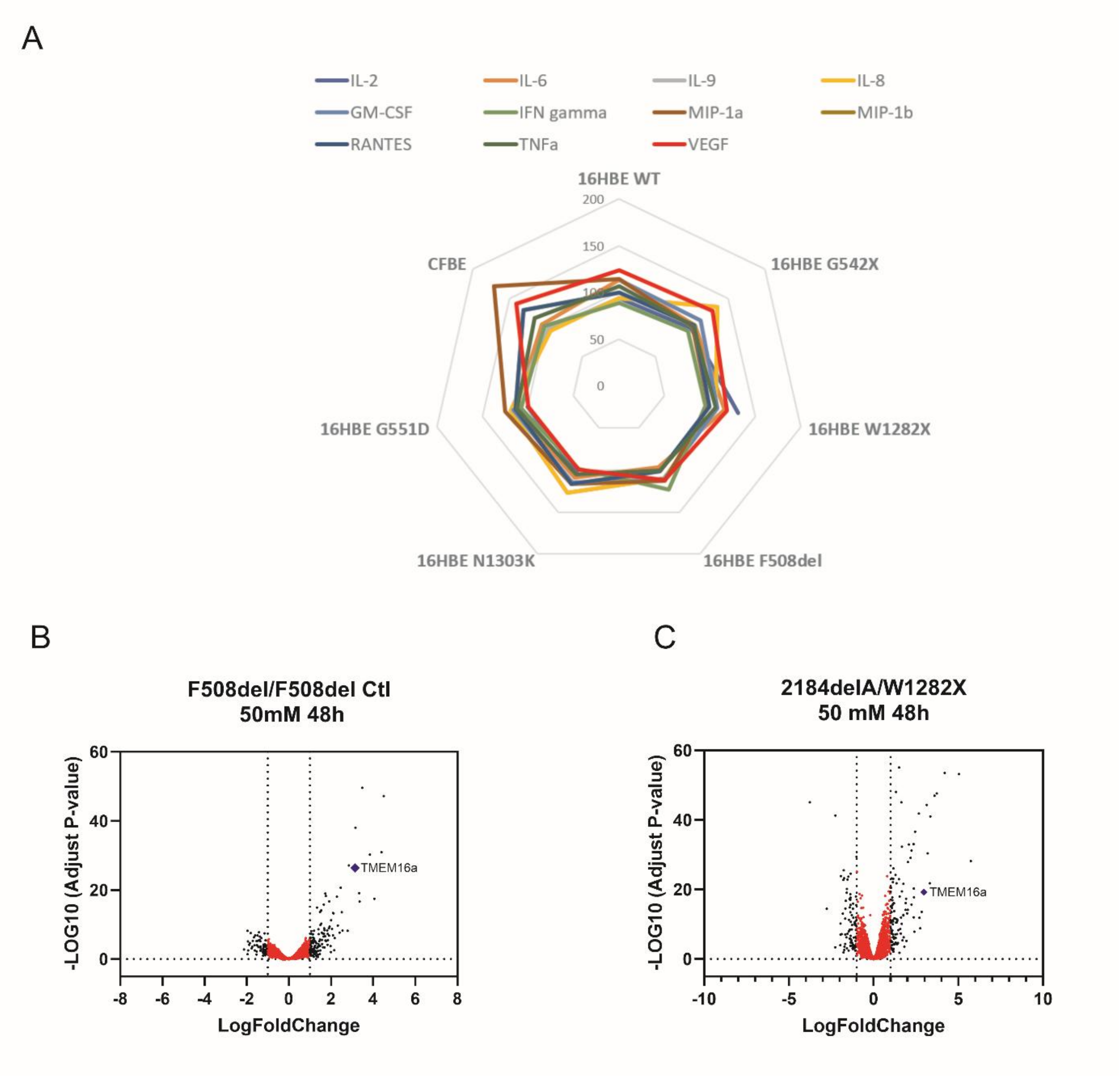

Fig. S1. TMEM16a ASO does not induce inflammation or toxicity.

A. After 24 hours of treating cells with either control ASO or TMEM16a ASO (50 nM), the media were collected, and cytokines were analyzed using multiplex analysis (Bio-Rad, France). The induction of cytokines was recorded in the control results for each cell line (n=5). No significant differences were observed in any of the cytokines tested. B. List of the various proteins analyzed using the in vitro profiling method. **B-C.** Volcano plot of transcriptomic analysis of hBEC cells cultured in ALI (n=5 cultures per patient). hBEC cells were obtained from a pwCF with class II mutations (F508del/F508del and 2184delA/W1282X). The cells

were treated with TMEM16a or Ctrl ASO (50 nM, 1h) for 3 days before mRNA extraction after a 48-hour incubation. **C-D** shows the top 10 mRNA fold changes observed after transcriptomic analysis, including the log of fold change and the -LOG10 P value.

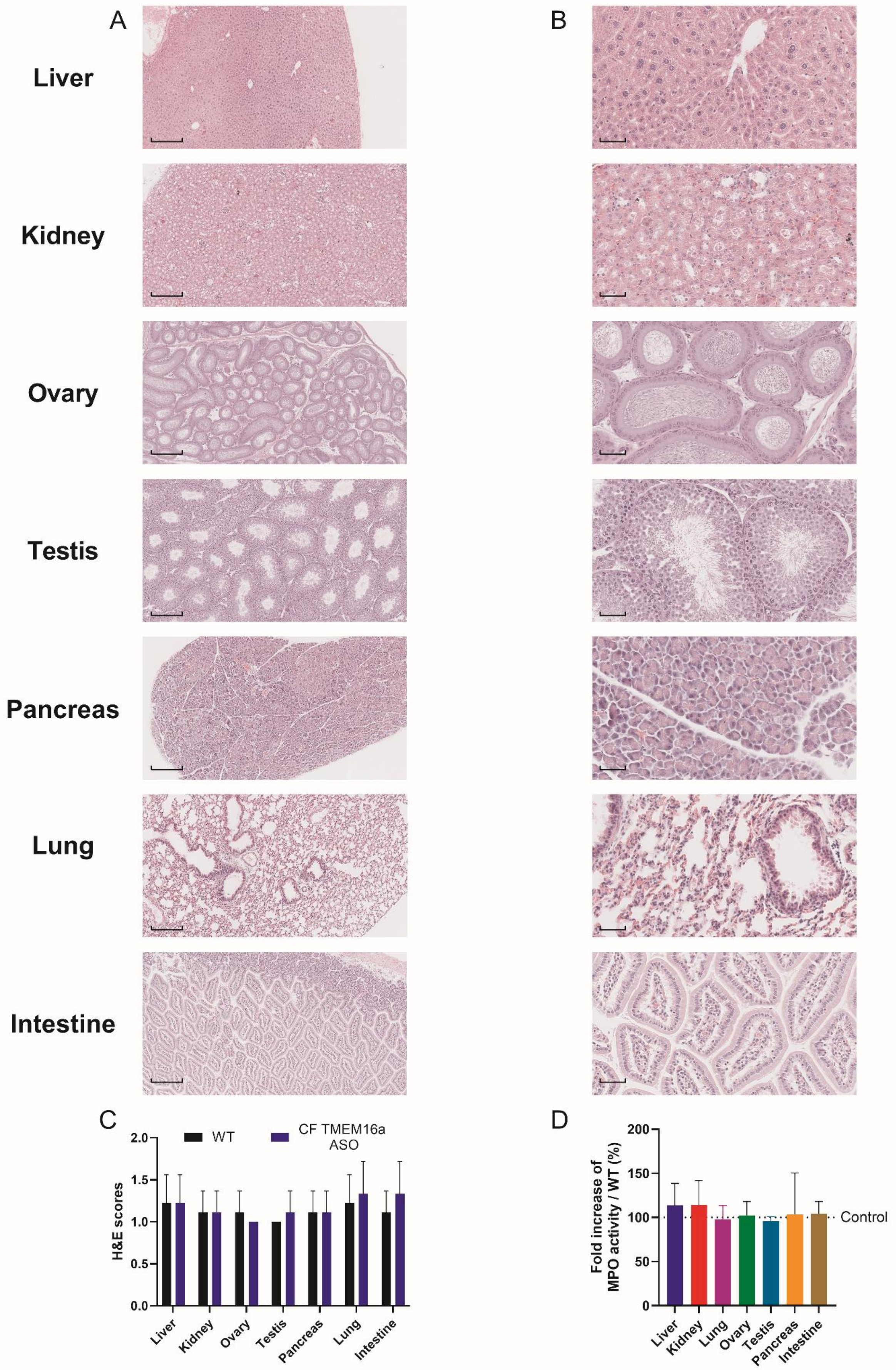
A
B
Liver
Kidney
Ovary
Testis
Pancreas
Lung
Intestine
C
WT
CF TMEM16a ASO
H&E scores
2.0
1.5
1.0
0.5
0.0
Liver
Kidney
Ovary
Testis
Pancreas
Lung
Intestine
D
Fold increase of MPO activity / WT (%)
200
150
100
50
0
Control
Liver
Kidney
Lung
Ovary
Testis
Pancreas
Intestine

Fig. S2. TMEM16a ASO does not induce histological effects on mice after one year of TMEM16a ASO treatment.

Representative histology of different tissues at 200x (**A**, scale bar 250µm) and 400x (**B**, scale bar 100 µm) magnification of the129-cftrtm1Eur CF mice. Following euthanasia after 1 year of treatment with TMEM16a ASO (10 mg/kg every 15 days). Mice were euthanized and mouse tissues (liver, kidney, ovary, testis, pancreas, lung, and intestine) were fixed in 4% paraformaldehyde, embedded in paraffin, sectioned (5µm), and subsequently stained with hematoxylin and eosin staining for histological analysis. **C** Semi-quantitative histological analysis of the H&E staining in the different tissues of CF mice treated with TMEM16a ASO for one year compared to untreated WT mice (n=9 per group). **D** MPO activity in different tissues. Values are expressed as mean values SD with a 95% confidence interval in fold of increase compared to tissues of WT mice. Statistical analysis was performed with ANOVA followed by Dunnett's and Bonferroni's post hoc tests. $^{**}p \leq 0.01$; $^{***}p \leq 0.001$; $^{****}p \leq 0.0001$,

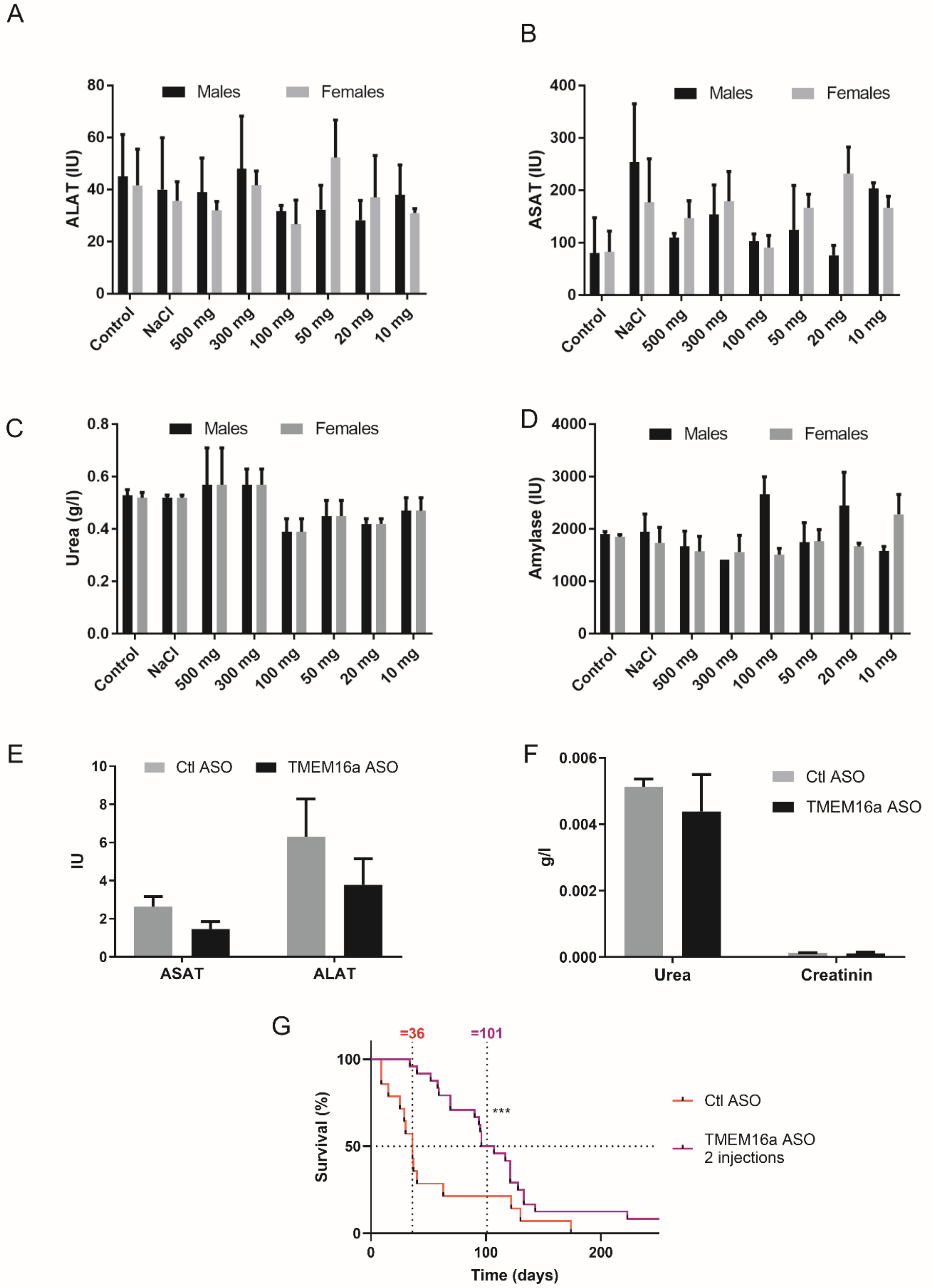
A
B
C
D
E
F
G
Males
Females
ALAT (IU)
ASAT (IU)
Urea (g/l)
Amylase (IU)
Control
NaCl
500 mg
300 mg
100 mg
50 mg
20 mg
10 mg
Ctl ASO
TMEM16a ASO
IU
ASAT
ALAT
g/l
Urea
Creatinin
=36
=101
***
Survival (%)
Time (days)
TMEM16a ASO
2 injections

Fig. S3. TMEM16a does not induce short or long-term toxicity.

A. Serum alanine aminotransferase (ALAT), **B.** Aspartate aminotransferase (ASAT), **C.** Urea, and **D.** amylase levels were determined in the blood of control and treated male and female mice (F508del/F508del) after injecting TMEM16a ASO subcutaneously at 0, 10, 20, 100, 300, and 500 mg/kg at 14 days post injection (n=3 /group). **E.** ALAT, ASAT, **F.** urea, and creatinine levels were determined 200 days after subcutaneous injection of TMEM16a ASO or control ASO (10 mg/kg) for long-term toxicity observations (n=5 /group). Data are represented as mean values ± SD, and Student's t-test was used to determine statistical significance. No significant difference was found. **G**. Kaplan-Meier survival curves of overall survival analysis in CF mice treated with only two injections of TMEM16a ASO at J11 and J18 (10 mg/kg) after birth, compared to mice treated with ASO control. Data are the mean of at least 15 mice per group. The dotted lines represent median survival when the staircase survival curve crosses 50%.

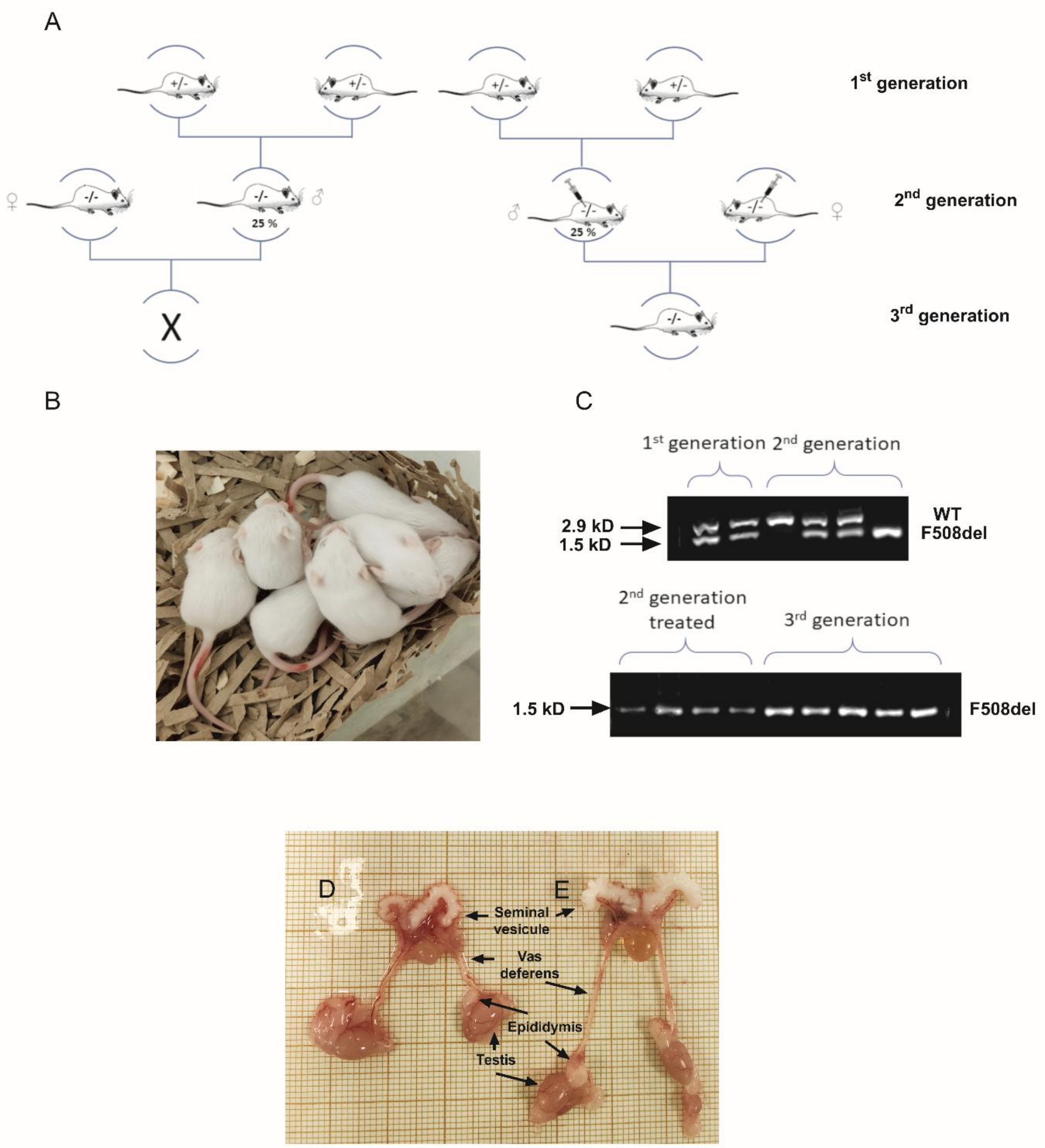

Fig. S4. TMEM16a ASO enhances fertility in CF male mice

Schematic representation of the reproduction of mice. In the first generation, heterozygote (-/+) mice were crossed to generate CF (-/-), WT (+/+), or -/+ mice. In the second generation, CF mice were crossed. Untreated TMEM16a ASO -/- male mice are not fertile (2nd generation, left panel). For treated mice, TMEM16a ASO was administered repeatedly by subcutaneous

injections (10 mg/kg) to parents (males and females) of the 2nd generation (right panel). Pups were born from CF-treated mice (3rd generation). **B**. Picture of an F508del/F508del litter born from a TMEM16a ASO-treated male and female CF mice. **C**. Representative genotyping gel after Ssp1 restriction enzyme digestion. This gel represents the normal breeding results (1st and 2nd generation) versus those of CF mice that are treated and can reproduce. **D-E.** Representative pictures of reproductive tissues of CF male mice after 4 weeks of control ASO **(D)** or TMEM16a ASO treatment **(E)**.
